# Organizational interventions in response to duty hour reforms

**DOI:** 10.1186/1472-6920-14-S1-S4

**Published:** 2014-12-11

**Authors:** Madelyn P Law, Elaina Orlando, G Ross Baker

**Affiliations:** 1 Department of Health Sciences, Brock University, St. Catharines, Ontario, Canada; 2 Institute of Health Policy, Management and Evaluation, University of Toronto, Toronto, Ontario, Canada

## Abstract

**Background:**

Changes in resident duty hours in Europe and North America have had a major impact on the internal organizational dynamics of health care organizations. This paper examines, and assesses the impact of, organizational interventions that were a direct response to these duty hour reforms.

**Methods:**

The academic literature was searched through the SCOPUS database using the search terms “resident duty hours” and “European Working Time Directive,” together with terms related to organizational factors. The search was limited to English-language literature published between January 2003 and January 2012. Studies were included if they reported an organizational intervention and measured an organizational outcome.

**Results:**

Twenty-five articles were included from the United States (n = 18), the United Kingdom (n = 5), Hong Kong (n = 1), and Australia (n = 1). They all described single-site projects; the majority used post-intervention surveys (n = 15) and audit techniques (n = 4). The studies assessed organizational measures, including relationships among staff, work satisfaction, continuity of care, workflow, compliance, workload, and cost. Interventions included using new technologies to improve handovers and communications, changing staff mixes, and introducing new shift structures, all of which had varying effects on the organizational measures listed previously.

**Conclusions:**

Little research has assessed the organizational impact of duty hour reforms; however, the literature reviewed demonstrates that many organizations are using new technologies, new personnel, and revised and innovative shift structures to compensate for reduced resident coverage and to decrease the risk of limited continuity of care. Future research in this area should focus on both micro (e.g., use of technology, shift changes, staff mix) and macro (e.g., culture, leadership support) organizational aspects to aid in our understanding of how best to respond to these duty hour reforms.

## Background

Patient safety concerns and interest in improving the work life of medical residents have led to duty hour reforms in a number of countries. Resident duty hour reforms and the European Working Time Directive have stimulated research to assess the impact of policy changes on clinical outcomes, patient safety, and resident training [1,2]. The internal organizational dynamics of health care settings are also being altered to compensate for these duty hour changes. However, to date, no systematic review detailing organizational interventions implemented in response to duty hour reforms is available to provide guidance for leaders and researchers. To that end, this paper summarizes reports on organizational interventions that have been made in direct response to duty hour reforms.

### Organizational challenges arising from reforms

The major emphases of research examining the consequences of duty hour reforms have been patient safety, resident training, and resident well-being. However, some of these studies have also examined aspects of organizational functioning affected by duty hour reforms, including costs [3-5], policy compliance [6-11], changes in work processes [12-17], and faculty satisfaction [14,18-20]. The majority of these studies relied solely on survey results related to staff perceptions of the impact of duty hour reforms; only a few assessed measurable, objective organizational outcomes [4,5,10,16].

Duty hour restrictions pose challenges for institutions for many reasons, including the need to restructure physician and other staffing to provide continued coverage for patients, the persistence of organizational cultures that promote working long hours and devalue compliance, and the financial implications of the restrictions. There are also concerns that reduced duty hours will limit residents’ ability to experience a broad range and number of clinical situations, reducing the value of their training experience. Further, duty hour reforms challenge the dominant professional cultures in some organizations, where working long hours is viewed as a “rite of passage” [21] and as a necessary means of gaining the range of experience needed to ensure effective training [22,23]. In fact, some organizations may permit residents to violate duty hour restrictions in order to gain experience or perform their work thoroughly [24]. These violations may conflict with management efforts to comply with operational, financial, and regulatory demands.

Grey literature reports from European Union (EU) countries have focused on variations between countries in the implementation of duty hour restrictions [25]. A report to the General Medical Council explained that duty hour restrictions have been difficult to implement and have created many organizational challenges. In that report, hospitals in the United Kingdom are reported to have a greater commitment to the culture of requiring long duty hours and are subsequently defying the new policy [21]. Studies from the United States show that the rates of individuals reporting non-compliance with the restrictions range from 13% and 18% [6,7] to 64.6%, 83.6%, and 90% [8,26,27].

Several studies have highlighted issues related to compliance with the duty hour restrictions. For example, in one US study, residents said they stayed at the hospital to complete their patient care tasks despite knowing they had exceeded the duty hour restrictions [24]. In another US study, 49% of residents admitted to under-reporting violations [27]. Non-compliance was also examined in an EU report on duty hours. It stated that in Ireland, for example, no hospitals were fully compliant with the provisions of the work time directive [25]. The study found that hospitals in countries where there has been a greater historical focus on work–life balance (e.g., France and Sweden) and where trainees have traditionally had a less prominent role in hospital care (e.g., Italy) have more easily integrated duty hour reforms [21].

One of the most salient organizational challenges has been the increased cost of implementing these reforms. Estimated costs in the United States for extra staff to cover the workload amount to between $1.1 and $1.6 billion per year [3,28]. Another US study estimated that transferring additional tasks to a lower-level provider would cost $673 million, and that using mid-level providers would cost $1.1 billion [29].

At an individual organizational level, one US study stated that hiring additional staff to implement program changes would cost $359,000 [4]. Numerous reports and comments from professional organizations have also claimed that staffing changes would increase costs, and that these increased costs may not be financially sustainable [30-32]. One report from the United Kingdom showed that the use of a consultant-delivered service would be more costly; however, this was thought to be balanced by better and faster decision making, thus reducing patient care costs [2].

In another report, however, Nuckols and Escarce [28] outline that there would be a cost saving for society if preventable adverse events were reduced by 2.4%, as well as a cost saving for major teaching hospitals if the preventable adverse event rates were reduced by 10.9%.

These reports illustrate the changes that health care organizations have had to make to their organizational processes and structures in order to respond to the duty hour reforms. Many organizations have been faced with new costs and have had to overcome cultural norms. These challenges require organizations to find and implement interventions that offer workable alternatives.

Given conflicting institutional demands and the influence of internal (i.e., staff and organization leaders) and external (i.e., regulatory bodies) actors on behaviour, how can an organization successfully implement the required duty hour reforms? To examine these responses, we reviewed research on organizational interventions, highlighting their results as well as opportunities for future research in this area.

## Methods

Our literature search focused on identifying articles that demonstrated a clear organizational intervention – with corresponding measures of organizational outcomes – implemented as a response to resident duty hour reforms and the European Working Time Directive (EWTD). The search focused on coupling “resident duty hours” and “European Working Time Directive” with specific organizational search terms as outlined in Table 1. Using the terms “resident duty hours” allowed for the identification of work in the United States, Canada, Australia, and other countries that referred to this issue using the term “resident duty hours.” “EWTD” was also used to capture articles from EU countries where terms other than “resident” are used to refer to doctors-in-training.

**Table 1:** Terms used in the literature search

Resident duty hours + European Working Time Directive +
quality of care organizational outcome organizational change staffing intervention system integration cost policy organizational implication efficiency effectiveness continuity of care innovation organizational consequence compromise institutional demands conflict staff shift handovers handoffs

The SCOPUS database, which includes MEDLINE, PubMed, and EMBASE, was used to search the literature. The search was limited to between January 2003 and January 2012. The year 2003 was used as a starting point because this was the first year in which Accreditation Council for Graduate Medical Education (ACGME) requirements were implemented [33]. The EWTD changes began in 2004, with full implementation in 2009 [2]. These activities prompted discussions and research studies exploring the outcomes of instituting these types of duty hour reforms. The initial search resulted in a total of 253 articles.

### Selection strategy

**Figure 1:**
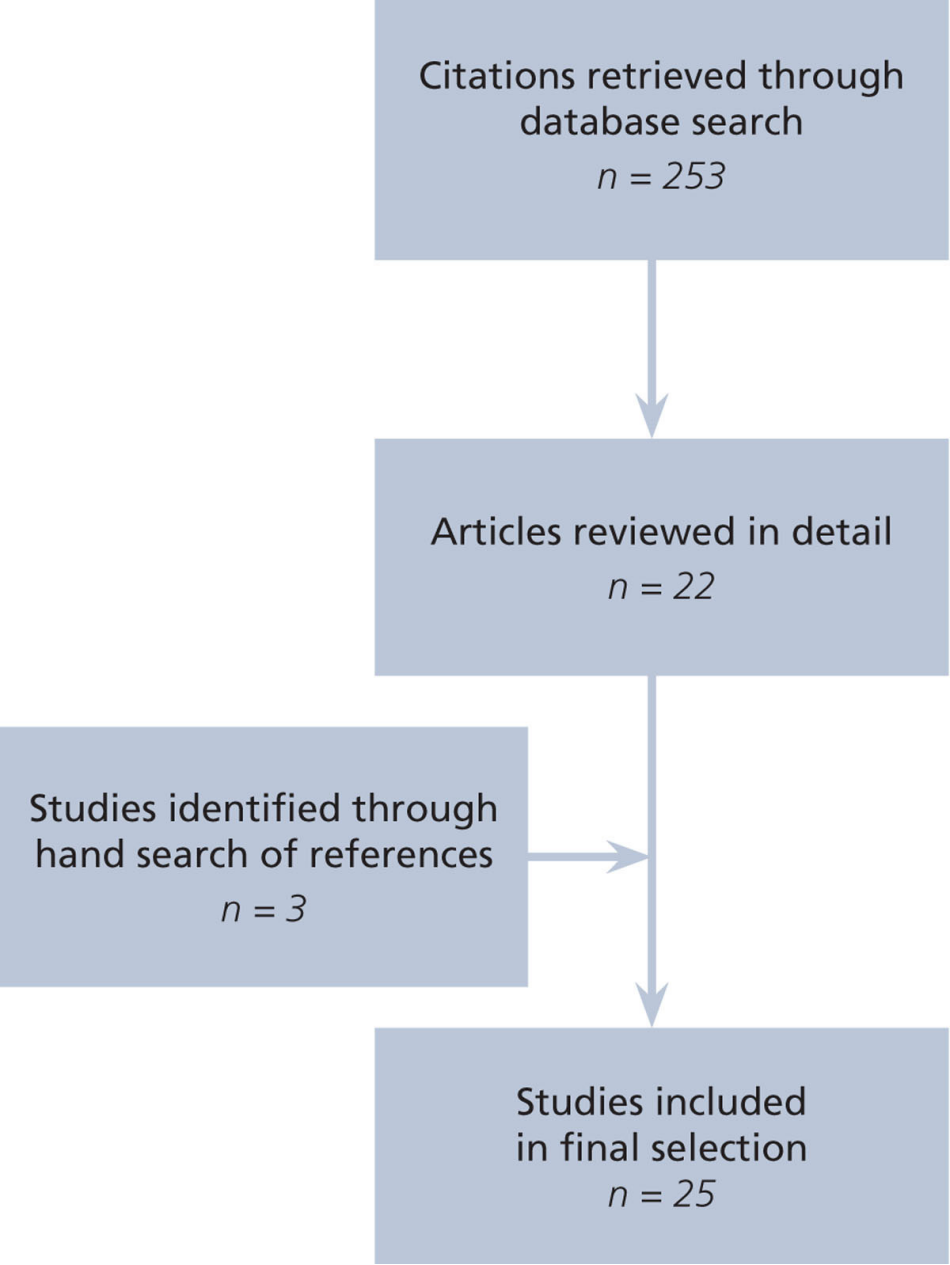
Flow diagram for literature search

Articles were deemed appropriate for inclusion if the research included a focus on an organizational intervention instituted in response to the organizational challenges created by duty hour reforms. An organizational intervention was defined “as a purposeful action by an agent to create and facilitate change in a particular organizational setting or system” [34,35]. For health care organizations, these interventions include changes in policy, practice, shift structure, technology, and personnel. Since other studies in this supplement focus on patient safety [36] and resident well-being [37], studies that focused *only* on patient outcomes and resident well-being were excluded. Articles were included if they provided measures and data describing health care improvement with respect to organizational factors such as costs, effectiveness, efficiency, compliance, coverage, and communications, and did so in a manner that went beyond speculative or cursory discussions (e.g., author discussion of perceived impact). From the initial search, 22 articles were identified that met these criteria. A hand search of the references in these articles identified three additional articles. This process resulted in a final sample of 25 articles (Figure 1).

### Data extraction

Upon completion of the initial review of abstracts, two of the authors (MPL, EO) further reviewed the full articles and extracted the data. See additional file 1 for the data used to perform the analysis that follows. (Although the focus of this paper is a discusson of organizational outcome measures, all other results (including individual- or patient-level) are also included in additional file 1.) The data was then assessed for completeness by the research team, who also discussed the findings and highlighted the overarching themes found in the articles.

## Results

Additional file 1 highlights the types of organizational interventions implemented in response to duty hour reforms. The majority of these studies were from the United States (n = 18), five were from the United Kingdom, one from Hong Kong, and one from Australia. All were single-site studies. Of these studies, the majority (n = 15) used a post-intervention survey to gather perceptions on organizational outcomes from residents, faculty, or other staff involved in the intervention [38-51]. Four studies used audit techniques to collect data on the measures [51-54]. Other studies used interviews [39], single-blind intervention [55], randomized crossover [56], impact evaluation [57], program evaluation [58], prospective self-controlled trial [59], data review [60], and retrospective cohort design [61]. Together these studies assessed organizational measures, including relationships among staff [39,40,62], work satisfaction [43,44,58], continuity of care [39,40,48,50], workflow [49,51,56], compliance [38,42,45,46,49,51,52,54,57,58,60], workload [40,41,43,52-54,59,60], and cost [51,61]. Interventions included using new technologies to improve handovers and communication [42,49,56], changing staff mixes [39-41,43,51,53,58,61], and implementing new shift structures [38,44-48,50-52,54,55,57-60], each of which had varying effects on the above-stated organizational measures.

### Technological interventions

A number of technologies were used to optimize communication between health professionals across shifts. These included a computerized multidisciplinary rounding and sign-out system [49], a computerized sign-out system [56], and a text- and voice-messaging system [42], all of which were implemented in hospitals in the United States. These systems were found to help improve workflow [49], enhance resident efficiency, and improve continuity of care [56], while at the same time increasing compliance with duty hour restrictions [42,49,56]. It should be noted that one study reported that implementation of a new system resulted in increased costs [42].

### Changes to staff mix

Several US studies described the use of nurse practitioners to accommodate the decrease in resident work hours. Nurse practitioners were reported as

• having a positive impact on resident education [39,40],

• enhancing or creating no change in the quality of care [40],

• reducing reliance on residents [43], and

• helping to deal with issues of compliance with resident duty hours restrictions [58].

Another US study reported recruiting a hospitalist to provide additional coverage and described improved satisfaction within teams and a decreased number of handovers as a result [62]. One hospital in the United Kingdom used clinical support workers and found that these workers reduced the amount of resident-provided direct patient care on certain tasks (e.g., cannulation, venepunctures) [53]. Various changes in the staff mix in hospitals in the United States were also identified as having a positive impact on the cohesion within units and with providing a positive experience among the team [40,62], increasing job satisfaction [43], and enhancing communication between residents and staff [39,41]. One study from the United States reported slightly reduced costs as a result of the intervention, which used physician assistants and hospitalists to accommodate the changes in staff coverage [61]. However, another hospital in the United Kingdom incurred increased costs as a result of implementing a modified multi-skilled night shift team [51].

### Innovative shift structures

Many hospitals have altered shift schedules as a result of duty hour reforms. One hospital in the United States using a day float system found that this change was viewed positively by residents and provided enhanced continuity of care for patients [48]. A variety of the shift models used in the United States, the United Kingdom, and Australia were also associated with improved compliance with the duty hour reform requirements [38,45,47,50-52,54,55,58]. One hospital in the United States redesigned its residency structure to include apprenticeship, small team, and night float models; these changes were associated with positive outcomes for residents who reported an increase in operative cases and stable or improved perceptions of caseload and continuity [57]. However, faculty in the same study reported increased work hours and job dissatisfaction as a result of the changes.

Another hospital in the United States developed a system aimed at ensuring compliance with the duty hour reforms that tracked individual residents’ work hours in real time. When a resident approached the weekly limit, he or she was dismissed for the required time the remainder of the week. This system was found to increase compliance with the restrictions, as well as the time off between shifts [60]. The use of a night-float system was also described in several studies as reducing the number of hours worked [41,54,59], although another intervention using a 16-hour shift and a resident in a night float resulted in no changes to the hours worked [46]. In one US study, an evening continuity clinic was implemented to replace a post-call clinic. This system was viewed positively by the residents, but was deemed to have a negative impact on the continuity of preceptors and access to medical services [44].

## Discussion

By examining the interventions reported in these studies, we can begin to understand how organizations are restructuring their work to respond to duty hour reforms. Many organizations are using new technologies, new personnel, and revised and innovative shift structures to compensate for reduced resident coverage and to decrease the risks associated with more limited continuity of care. However, it should be noted that the studies examined were all based at individual sites, and so their results may reflect a variety of factors that are not controlled for. As such, the success of these interventions may rest on more than just the extent to which new policies and work processes provide adequate staff coverage. As well, the majority of the studies were conducted in academic teaching hospitals, with eight of the interventions focused on surgical units/departments and seven on internal medicine. Most were implemented in single units or departments within a hospital, rather than hospital wide. Comparisons across these studies is difficult, given that the studies did not all provide the level and type of information that would allow for generalizations to other organizational contexts.

A number of the studies examined reported on outcomes related to patient safety, as well as organizational changes. For example, no significant differences in the number of reported incidents were attributable to a computerized rounding and sign-out system [56]. In addition, no change in patient mortality was associated with a number of described interventions [38,47,57], and no change in length of stay was seen in the one study that incorporated a shift structure intervention [38]. Another study found a perceived improvement in communication between residents and nurses after the implementation of a night-float system [41]; this could also be seen to contribute to improved patient safety. Another study found a reduction in handovers; this could also have a positive impact on patient safety [62]. Differences were seen in the continuity of care in a study reporting changes in shift structure, in which 80% of respondents perceived a decline in patient care [50]. However, two studies outlined better continuity of care arising from shift structure changes [48] and changes in staff mix [39,40].

Duty hour reforms have both “technical” and “adaptive” components [63]. The changes not only require modifications to staffing responsibility and team composition, but also challenge underlying assumptions about what constitutes effective coverage and effective medical education. The considerable variation reported in compliance with duty hour reforms suggests varying responses to the new policies. These variations may also have a bearing on the successful implementation of new staffing structures and policies. Organizations in which medical leadership and staff perceive duty hour reforms as a challenge to effective residency experiences might be less successful in implementing new staff mixes or schedules. In contrast, those organizations that accept the need for reduced duty hours and seek ways to compensate for increased handovers may be more successful in altering their current practices.

The current literature considers neither the extent to which organizations have embraced the need for reforms, nor the underlying cultural dynamics that may contribute to their acceptance. Focusing narrowly on interventions that target compliance and workload coverage, although an important stepping stone, does not address the need to create an organizational culture in which duty hour reforms are valued and supported by staff. Examining the organizational culture and leadership attributes and actions that support reforms and assist successful implementation of these policies and practices may provide a more detailed understanding of why and how these interventions succeed in some contexts but not in others [64,65].

Future research in this area should focus on both technical (e.g., use of technology, shift changes, staff mix) and adaptive (e.g., culture, leadership support) organizational aspects to aid in our understanding of how best to respond to these duty hour reforms. Pache and Santos [66] note that the way in which organizations respond to conflicting institutional demands varies greatly depending on the organization’s internal actors. Additional studies that examine the broader context supporting new staff mixes, shift structures, and communication technologies would likely provide an informative lens through which to understand successes and challenges in specific organizations and, ultimately, to allow for a deeper appreciation of the lessons learned.

## Conclusions

The research on the organizational impact of duty hour reforms is limited, and the majority of the work stems from the reported experiences of individual hospitals in the United States. Further research is needed to identify the broader organizational attributes that may contribute to success or failure in creating and implementing organizational change. This knowledge will help hospitals assess how interventions used in other settings might be translated into local practice and how, ultimately, to select and implement their own interventions.

## Authors' contributions

The authors contributed equally to this work. MPL participated in the the literature search and the data review and synthesis, and led the development of the manuscript. EO participated in the literature search and data synthesis and helped to draft and edit the manuscript. GRB participated in the data analysis and the writing and revision of the final paper. All authors read and approved the final manuscript.

## Competing interests

The authors declare that they have no competing interests.

## Supplementary Material

Additional file 1 The impact of interventions used in response to changes in resident duty hours Click here for file

## Declarations

Resources and secretariat support for this project was provided by the Royal College.

This article has been published as part of *BMC Medical Education* Volume 14 Supplement 1, 2014: Resident duty hours across borders: an international perspective. The full contents of the supplement are available online at http://www.biomedcentral.com/bmcmededuc/supplements/14/S1. This article was submitted and peer reviewed in 2012. Final acceptance for publication as part of this supplement was in 2014.

Publication of this supplement was supported and funded by the Royal College of Physicians and Surgeons of Canada. The funding agency played no role in the design, in the collection, analysis and interpretation of data; in the writing of the manuscripts; and in the decision to submit the manuscript for publication. The articles have been through the journal's standard peer review process for supplements. The Supplement Editors declare that they have no competing interests.
