# Supplementary material for: Organizational interventions in response to duty hour reforms
Source: BMC Med Educ. 2014 Dec 11;14(Suppl 1):S4. doi: 10.1186/1472-6920-14-S1-S4 (PMC4304281; doi:10.1186/1472-6920-14-S1-S4)
Supplement: Additional file 1 — The impact of interventions used in response to changes in resident duty hours [file 1472-6920-14-S1-S4-S1.doc]

## Organizational interventions in response to duty hour reforms

## Additional file 1: The impact of interventions used in response to changes in resident duty hours

| **Study** | **Study design** | | **Intervention** | **Context** | **Focus of assessment and measures** | **Organizational impact** |
| --- | --- | --- | --- | --- | --- | --- |
| ***Technology interventions*** | | | | | | |
| Goldstein et al. (2005) [60] | Review of work hour data from web-based database | | Work Hours Assessment and Monitoring Initiative (WHAMI) | Department of Surgery; New York Presbyterian Hospital, Columbia Campus; New York, US  913-bed tertiary care centre; advanced and specialized surgical care; primary care for local urban community  Trains 52 residents and three physician assistants | Work hours; compliance | Compliance with duty hour requirements increased by 93% to 99% |
| Goldstein et al. (2009) [42] | Pre- and post-survey of surgical residents | | Implementation of an IT system, using text messaging and short-message systems, to enhance compliance | Department of Surgery; Maimonides Medical Center; New York, US | Feasibility of system integration; compliance with duty hour restrictions | Increased costs were required to support the system; residents reported an increase in ability to comply with duty hour regulations from 47% to 74% |
| Van Eaton et al. (2010) [56] | Randomized crossover design study of inpatient resident teams at two hospitals | | Computerized rounding and sign-out system (“UW Cores”) | Harborview Medical Center* and University of Washington Medical Centre,† Washington, US  ^*^368-bed adult and pediatric trauma centre  ^†^ 450-bed tertiary care academic hospital | Resident reported deviations from expected care per 1,000 patient-days; number of incidents, medical errors; time spent copying patient data, resident efficiency | No difference between groups in mean number of resident-reported deviations from expected care per 1,000 patient-days; no significant differences in number of reported incidents, odds of a medical error |
| Wohlauer, et al. (2012) [49] | Pre–post self-administered questionnaire | | Computerized multidisciplinary rounding and sign-out program to automate collection of clinical information | Surgical residency program; University of Colorado; Colorado, US | Workflow | Improved resident workflow efficiency; 70% of residents reported new program helped achieve compliance with the 80 work week |
| ***Innovative shift structures*** | | | | | | |
| Afessa et al. (2005) [38] | Survey of attending intensivists, critical care fellows, first- and third-year internal medicine residents | | 14-hour shift model of rotation for internal medicine residents and critical care fellows | MICU; tertiary referral centre; US  24-bed unit | Patient outcomes; work hours | Residents were able to comply with duty hour requirements; no change in patient mortality rates or length of stay |
| Cappuccio et al. (2009) [55] | Single-blinded intervention study | | Implementation of European Working Time Directive (EWTD) – compliant 48-hour work week over a 12-week period | Critical Decision Unit, Care of the Elderly, Endocrinology & Respiratory Units; University Hospitals Coventry & Warwickshire NHS Trust; Coventry, UK  1250-bed hospital | Impact on patient safety; doctors’ work–sleep patterns | Intervention reduced the average duration of the schedule work shifts by nearly one hour |
| Cass et al. (2003) [52] | Pre- and post-work audit | | Change to full-shift night team, including three middle-grade doctors and two senior nurses; development of new staff roles | UK  Large children’s hospital | Compliance with duty hour regulations; workload | Minimal change in workload was found; compliance with duty hour regulations increased by 33% to 77% |
| Garvin et al. (2008) [50] | Survey of general surgery senior house officers (SHOs) | | Implementation of a pilot EWTD-compliant rota | Department of General Surgery; Galway University Hospital; Ireland, UK | Compliance with EWTD; perceived impact on continuity of care; impact on training; impact on quality of life | SHOs felt that continuity of care was not achieved 70% of the time; 81% perceived a decline in patient care |
| Goldstein et al. (2004) [41] | Retrospective survey of general surgery residents | | Use of a dedicated night-float system | Department of Surgery; New York Presbyterian Hospital, Columbia Campus; New York, US  1000-bed tertiary care center; advanced and specialized surgical care; primary care for local community | Work hours; resident well-being | Average weekly work hours were reduced by 25%; there was a perceived improvement in communication between residents and nurses; hospital complaints were reduced by 27% |
| Lee et al. (2003) [59] | Prospective self-controlled trial of surgical residents before and after implementation of intervention | | Use of a night shift call system | United Christian Hospital; Hong Kong  245 surgical bed district hospital  Small training program | Number of hours worked | There was a significant reduction in the consecutive number of hours worked by surgical trainees |
| Mathis et al. (2006) [45] | Pre- and post-intervention survey of residents and faculty | | Implementation of a ward call team system for one year | Internal medicine residency program at University Hospital at the University of Cincinnati Medical Center; Cincinnati, US  650-bed urban, academic, teaching hospital; admits approximately 14,000 patients annually | Perceived impact on duty hour requirements and patient care | Faculty perceived an improved compliance with duty hour requirements and were neutral regarding impact on patient care; residents were neutral on both topics |
| McCoy et al. (2011) [46] | Pre- and post-survey of gastroenterology teams | | Implementation of 16-hour shifts, with one resident assigned to a night shift every other night for 12 days; the remainder of the month consisted of day shifts | Mayo Clinic, Minnesota; US | Patient care, education, duty hour compliance | No change in the number of hours worked; residents felt they were less prepared to handle cross-coverage issues |
| Ogden et al. (2006) [62] | Survey of residents | | Implementation of a call system whereby the primary call team takes admissions from 12 p.m. to 8 p.m. daily, leaves by 10 p.m. and returns after 10 hours for a full post-call day; after hours admissions are managed by hospitalists | Internal medicine residency program; Texas A&M / Scott & White Memorial Hospital; Texas, US  490-bed, acute care hospital; primary teaching hospital.  14 categorical residents per year and 6 preliminaries. | Residents experience on general medicine inpatient teaching (GMIT) teams; impact on rounds and inpatient teaching, number of handoffs; residents’ perception of impact on education, patient care, and personal life | Residents reported an improvement in experiences on GMIT teams and a reduction in handoffs |
| Roey (2006) [47] | Survey of residents to assess impact of new system on educational activities | | Day float team responsible for new patient admissions in a.m. | Santa Clara Valley Medical Center inpatient medical service; California, US  528-bed county hospital affiliated with Stanford University School of Medicine  Admits approximately 800 to 900 patients monthly; no separate specialty services | Education and resident work life | House staff reported increased autonomy, enhanced teaching from attending |
| Schneider et al. (2007) [57] | Impact evaluation over two years | | Creation of small team rotations, apprentice rotations, elective rotations, and night float rotations | Northwestern University, Feinberg School of Medicine, Department of Surgery; US | Number of operative cases; compliance with ACGME requirements for volume and distribution | Modified program structure was found to be associated with positive increases in resident perceptions of various aspects of their rotations; no significant changes in patient monthly mortality or number of admissions |
| Suryadevera et al. (2008) [48] | Survey of otolarynology residents | | Implementation of a day-float coverage system allowing residents to participate in a 24-hour call period | Otolarynology department, linked university, veterans, and private hospitals; US | Resident experiences and perception of system | Residents perceived that the day-float system enables continuity of care |
| ***Changes in staff mix*** | | | | | | |
| Bahouth et al. (2007) [39] | | Interviews and survey with 58 nurse practitioners (NPs) plus interviews with residents, attendings, and nursing staff | Expanded role of nurse practitioners in Academic Medical Centers (AMCs); number of NPs increased from 38 to 73 from 2002 to 2006 | University of Maryland Medical Center; US  743-bed AMC; highly specialized tertiary and quaternary care  787 residents and fellows,  several training programs | Relationships between house staff and nurse practitioners; education; continuity of care | Surgical residents reported that NPs provided greater continuity in care allowing them to focus on operating room time and education |
| Buch et al., (2008) [40] | | Survey of residents and non-physician practitioners (NPPs) | Integration of NPPs within surgical subspecialty teams | Surgical residency program; Mount Sinai; US  72 residents | Relationships between residents and NPPs; educational impact; continuity of care; workload; communication; collaboration; roles | Residents felt that NPPs reduced their workload; significantly more NPPs felt they contributed to the clinical education in comparison with residents (75.0% vs. 38.5%, *p* = .005); significantly more NPPs than residents felt they provided better continuity of care compared to only 38.5% of residents (96.4% vs. 60.6%, *p* = .005) |
| Herbertson et al. (2007) [53] | | Audit at two points in time | Addition of a clinical support worker (CSW) | Nottingham City Hospital; UK | Workload | The CSW helped to reduce the workload on specific tasks for junior doctors |
| Holleman, et al. (2010) [43] | | Survey of physicians, nurses, and allied care practitioners | Addition of pediatric NP to pediatric neurosurgical academic program | University of Chicago Children’s Hospital; US | Impact on provider satisfaction; patient clinical satisfaction; workload | Workload was reduced during NP hours; significant change in all satisfaction measures including availability, responsiveness, and patient clinical satisfaction |
| Jones et al. (2004) [51] | | Survey; work hours audit | Modified focus of care to consultant-led, specialist registrar (SPR) service, use of multi-skill night-shift team and replacement of on-call shifts with full shifts | Royal Free Hampstead NHS Trust; UK | Compliance with EWTD; impact on clinical care and training opportunities; cost; workload | Compliance increased from 62% to 90%; significant costs associated with lost outpatient activity because of specialties contributing an SPR to night teams |
| Kuo et al. (2004) [44] | | Two time-point follow-up survey of pediatric residents | Replacement of post-call continuity clinics with evening continuity clinics | San Francisco General Hospital; California, US  Medical care for many ethnic minorities, under and uninsured populations.  23 residents; second-largest clinic site in the program | Resident satisfaction and experience with evening clinics compared to post-call clinics | Continuity of preceptors and access to medical services were perceived to be worse |
| Lundberg et al. (2006) [58] | | Program evaluation | Assignment of an acute care NP to each post-call team to enable resident sign-out by 2 p.m. | Olive View UCLA Medical Center; US  One of four acute care hospitals in the Los Angeles County Department of Health Services; 130,000 scheduled visits yearly to outpatient clinics; 48,000 unscheduled patient emergency department visits yearly; internal medicine service admits approximately 24 patients daily  Eight teams: one attending, one senior resident, and two first-year residents | Compliance with ACGME duty hour restrictions; physician satisfaction | Compliance with ACGME requirements was found to be 99%; average hours worked per week decreased for inpatient ward residents from 84 hours to 76 hours; physicians and residents were highly satisfied with the addition of the NP, with a rating of 8.8 out of 9 on a satisfaction scale |
| Roy et al. (2008) [61] | | Retrospective cohort | Use of localized services with physician assistants supervised by hospitalists | Inpatient general medicine; urban, academic hospital; Northeastern US  747-bed academic medical centre | Length of stay; cost of care; mortality; ICU transfers; readmission rate; patient satisfaction | Length of stay was similar to traditional house staff; cost of care was slightly less for the intervention group; there were no significant differences in readmissions, ICU transfers, mortality, or patient satisfaction |
| Wysocki & McGowan (2010) [54] | | Audit at two periods | Impact on workload of changes to rostering and structure of a surgical unit | Department of Surgery; Logan Hospital; Queensland, Australia | Total number of hours worked; compliance with duty hour regulations; fatigue as measured using the Fatigue Audit InterDyne system | The number of hours worked decreased by 11% post restructuring; compliance increased from 67.3% to 91.2%; fatigue decreased from 133.25 hours to 0 hours |
